# Xanthoceraside Ameliorates Mitochondrial Dysfunction Contributing to the Improvement of Learning and Memory Impairment in Mice with Intracerebroventricular Injection of A**β**1-42

**DOI:** 10.1155/2014/969342

**Published:** 2014-05-25

**Authors:** Xue-Fei Ji, Tian-Yan Chi, Qian Xu, Xiao-Lu He, Xiao-Yu Zhou, Rui Zhang, Li-Bo Zou

**Affiliations:** Department of Life Science and Biopharmaceutics, Shenyang Pharmaceutical University, Shenyang 110016, China

## Abstract

The effects of xanthoceraside on learning and memory impairment were investigated and the possible mechanism associated with the protection of mitochondria was also preliminarily explored in Alzheimer's disease (AD) mice model induced by intracerebroventricular (i.c.v.) injection of A*β*1-42. The results indicated that xanthoceraside (0.08–0.32 mg/kg) significantly improved learning and memory impairment in Morris water maze test and Y-maze test. Xanthoceraside significantly reversed the aberrant decrease of ATP levels and attenuated the abnormal increase of ROS levels both in the cerebral cortex and hippocampus in mice injected with A*β*1-42. Moreover, xanthoceraside dose dependently reversed the decrease of COX, PDHC, and KGDHC activity in isolated cerebral cortex mitochondria of the mice compared with A*β*1-42 injected model mice. In conclusion, xanthoceraside could improve learning and memory impairment, promote the function of mitochondria, decrease the production of ROS, and inhibit oxidative stress. The improvement effects on mitochondria may be through withstanding the damage of A*β* to mitochondrial respiratory chain and the key enzymes in Kreb's cycle. Therefore, the results from present study and previous study indicate that xanthoceraside could be a competitive candidate for the treatment of AD.

## 1. Introduction

Alzheimer's disease is a progressive neurodegenerative disease. As the most common form of irreversible dementia, it is forming an increasing and considerable burden on patients, families, and society [1]. AD is characterized by *β*-amyloid (A*β*) deposition, neurofibrillary tangles, synaptic loss, and selective loss of neurons with the clinical features of recognition impairment, memory damage, and personality changes. A*β* is a self-aggregating peptide produced by cleavage of a transmembrane amyloid precursor protein (APP) [2, 3]. The senile plaque extracellularly formed by A*β* fibrils was one of the hallmarks of AD [2, 4]. It is widely recognized that A*β* fibrils exhibited neurotoxicity contributing to the progress of AD. In contrast to original thinking, recent studies showed that soluble A*β* oligomers displayed more potent neurotoxicity since they had a better correlation with AD severity. Moreover, more and more research proposed the intracellular toxic mechanism of A*β* [5, 6]. Overall, A*β* aggregation and the resulting cascade prompted the progress of AD.

Currently, many researchers assumed mitochondrial dysfunction as the early event in AD development and the toxicity of A*β* to mitochondria have been attracting more and more attention of the researchers. Mitochondria are dynamic organelles providing most of the cellular ATP demand by oxidative phosphorylation, maintaining Ca^2+^ homeostasis, and taking part in apoptosis [7–9]. Mitochondrial dysfunction has been found in brains of both AD patients and AD transgenic mouse models, as well as in cell-lines expressing mutant APP or treated with A*β* [8–10]. It is by far reported that mitochondrial abnormality occurs during the AD process and contributes to its onset and progression; however, whether mitochondrial deficits are the primary cause of the neuronal damage or a secondary response caused by other pathologies is an unresolved issue by now. Studies found selective reduction of mitochondrial cytochrome c oxidase (COX) activity with age and in postmortem AD brains [11, 12]. Chronic injection of sodium azide (NaN_3_), inhibition of COX, caused cognitive deficit in rats. Some studies proposed that COX deficiency drives increased oxidative damage and predisposes for the formation of A*β* [13], while others denied this notion and concluded that COX defect is rather a consequence of A*β* [14, 15]. Consistently, A*β* may affect the enzyme activity in mitochondrial inspiratory chain or Kreb's cycle, such as PDHC (pyruvate dehydrogenase complex), KGDHC (*α*-ketoglutarate dehydrogenase complex), and ABAD (A*β*-binding alcohol dehydrogenase) [8, 13, 16]. The ROS (reactive oxygen species) production induced by A*β* may lead to the deleterious mitochondrial function and result in ROS increase to form a vicious circle. Moreover, A*β* may induce mutations of mitochondrial DNA and RNA or influence the mitochondrial dynamics [17, 18].

**Figure 1:**
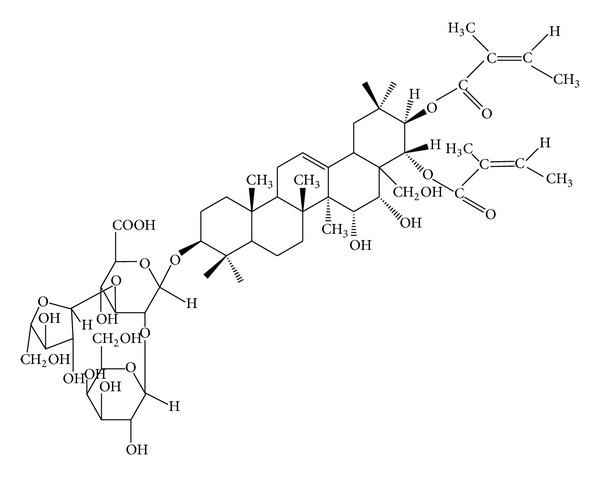
The chemical structure of xanthoceraside.

*Xanthoceras sorbifolia* Bunge (XSB) is a special woody oil plant which is given priority to develop as bioenergy plant in 21st century in China. The seeds of XSB are the raw material to produce biodiesel; however, the husk of XSB usually is discarded as processing remains, which is a huge waste of resources. The medical benefits of XSB have been recorded since ancient time in China and extracts of XSB seeds have been developed as the drug to treat pediatric enuresis in recent years. Based on the fact above, we did a series of research about xanthoceraside (Figure 1), a triterpene extracted from the husks of XSB, on its antiamnesic and neuroprotective effects. Our results showed that xanthoceraside had significant protective effects on learning and memory impairment in several AD animal models such as A*β*25-35 induced AD mice model [19, 20]. The mechanism may involve antioxidative stress and antiapoptosis. However, it is unclear whether improvement of mitochondrial function is involved in the ameliorative effects of xanthoceraside on learning and memory impairment. In the present study, we used water maze test and Y-maze test to confirm the effects of xanthoceraside on learning and memory impairment induced by aggregated A*β*1-42. We specially examined its effect on mitochondrial function including the ATP level, ROS level in cerebral cortex and hippocampus in mice, and activities of key mitochondrial related enzymes in isolated cerebral cortex mitochondria.

## 2. Materials and Methods

### 2.1. Materials

Xanthoceraside was provided by Shenyang Institute of Applied Ecology, Chinese Academy of Sciences (Shenyang, China), and its chemical structure is shown in Figure 1. It was dissolved in distilled water for oral administration by gavage. Amyloid *β*-protein 1-42 (A*β*1-42) was purchased from Sigma (St. Louis, MO, USA). Huperzine A (Shanghai Fuhua, China) was used as positive control in this study.

### 2.2. Animals

Male Kunming mice (6 weeks old) were obtained from Changsheng Biotechnology Co., Ltd, Liaoning, China. The mice were housed in polyacrylic cages (5 mice per cage) under individual ventilated caging (IVC) system, and they were kept in a regulated environment (23 ± 2°C, 50 ± 5% humidity) with a 12/12 h light/dark cycle. Food and water were provided ad libitum. All the animal studies were performed in strict accordance with the guiding principles for the care and use of laboratory animals in China and the guidelines established by the Institute for Experimental Animals of Shenyang Pharmaceutical University. All efforts were performed to minimize the suffering of the animals throughout the study. Behavioral experiments were carried out in a sound-attenuated and air-regulated experimental room, in which mice were habituated beforehand for at least 1 h.

### 2.3. Treatment

A*β*1-42 was dissolved in sterile physiological saline and aggregated by incubating at 37°C for 5 days before the injection. The mice were anesthetized with chloral hydrate (300 mg/kg i.p.) and placed on a stereotaxic apparatus with a mouse adaptor. 3 *μ*L aggregated A*β*1-42 (≈410 pmol/mouse) was injected into the right cerebroventricle (−0.5 mm anteroposterior, 1.0 mm mediolateral from bregma, and −3 mm dorsoventral to dura) according to the atlas in [21] on day 0. Control animals were injected with equivalent volumes of vehicle by the same operation [22, 23]. On day 1, the A*β*1-42 injected mice were divided into 5 groups, xanthoceraside-treated groups (0.08, 0.16, or 0.32 mg/kg), huperzine A-treated group (0.039 mg/kg), and model group (distilled water), and they were treated by gavage once daily until the last day of the study. Each group consists of 12 mice. The three doses of xanthoceraside were determined according to our previous study [20].

### 2.4. Morris Water Maze Test

The Morris water maze test was performed from day 8 to day 11. The apparatus consisted of a round water pool (100 cm in diameter and 50 cm in height) and a removable platform. The water temperature was controlled at 22°C ± 1°C and the depth was 30 cm. The platform (10 cm in diameter) was placed 1 cm below the water surface in the fourth quadrant [20, 24]. Mice were trained two trials a day for four consecutive days with an intertrial interval of 3 h. During the training period, each mouse was gently placed at one of the two different positions in the first quadrant and allowed to swim escaping onto the platform, and the escape latency (the spending time to find the platform) was recorded for 60 s. After 60 s, if the mouse failed to locate on the platform, it was then placed on the platform for 10 s stay. On the fourth day, the mice were given a probe test, in which the platform was taken away, and each mouse was placed at one point in the first quadrant. The mouse was allowed to explore in the water pool for 60 s. The swimming time and the swimming distance in the fourth quadrant were calculated. In this test, the swimming trails of each mouse were tracked by a video camera and the data were recorded automatically with computer software (designed by Institute of Materia Medica, Chinese Academy of Medical Science).

### 2.5. Spontaneous Alternation in the Y-Maze

The Y-maze test was performed on day 12. The Y-maze apparatus consisted of three identical arms (each arm was 40 cm long, 12 cm high, 5 cm wide at the bottom, and 10 cm wide at the top) which were converged at the central equilateral triangle area to allow the mice to freely enter into any of the arms. Mice were placed at the end of one arm and allowed to explore freely for 5 min. The sequence of arm entries was recorded. Alternation was defined as entering into three different arms consecutively. The spontaneous alternation behavior (%) was calculated according to the following formula [3, 19]:

(1) Spontaneous alternation behavior (%) = number of alternationtotalnumber of arm entries−2×100%

### 2.6. ATP Levels

After the decapitation, the cerebral cortex and hippocampus of mice were rapidly dissected on ice, followed by adding a suitable volume of lysate, respectively. The tissue solutions were homogenized using an ultrasonic crusher and centrifuged at 18000 g for 20 min at 4°C. The supernatant was collected and used for the determination of ATP level with an ATP assay kit utilizing a microplate luminometer (Centro XS3 LB960, Berthold Technologies).

### 2.7. ROS Levels

The cerebral cortex and hippocampus of the mice were dissected individually and minced in 2 mL medium 1 (NaCL 138, KCL 5.4, Na_2_HPO_4_·12H_2_O 0.22, glucose 5.5, and sucrose 58.4 all in mmol/L, PH 7.35), further dissociated by trituration through a mesh (pore diameter 75 *μ*m). The dissociated cells were washed twice in medium 2 (NaCL 110, KCL 5.3, CaCL_2_ 1.8, MgCL_2_ 1, glucose 25, sucrose 70, and HEPES 20 all in mmol/L PH 7.4) through centrifugation at 1000 g for 3 min at 4°C. After washing, the cells were resuspended in 6 mL DMEM and 250 *μ*L aliquots were added to black 96-well plates followed by incubation with fluorescent probe H_2_-DCFDA (final concentration 10 *μ*mol/L) for 30 min at 37°C. After washing twice with Hank's balance solution (KCL 5.33, KH_2_PO_4_ 0.44, NaHCO_3_ 4, NaCL 138, and Na_2_HPO_4_·12H_2_O 0.34 all in mmol/L) [25], the formation of the fluorescence was detected at 485 nm excitation/535nm emission using a Thermo Scientific Varioskan Flash microplate reader.

### 2.8. Mitochondria Isolation

Mice brain mitochondria were isolated by Percoll density gradient centrifugation method described by Sims and Anderson [26–28] with a number of major modifications. Briefly, on day 13, the mice were decapitated and the cerebral cortex tissues were rapidly removed (within 1 minute) to ice-cold isolation buffer containing 225 mM mannitol, 75 mM sucrose, 1 mM EGTA, 0.2% BSA, and 20 mM HEPES (PH 7.4). The tissues were finely minced with scissors and homogenized by hand in all-glass homogenizer, followed by centrifugation at 2000 g for 4 min. The supernatant was collected and centrifuged at 12000 g for 10 min producing a loose pellet as crude mitochondria. The pellets were resuspended in 15% Percoll and layered on 25% Percoll above 40% Percoll to form discontinuous density gradient, which were centrifuged at 30000 g for 10 min. The fraction accumulating near the interface of the lower two layers was collected and washed once to acquire pure mitochondria. All the steps above should be performed on ice. Mitochondria protein content was determined by the Bradford assay.

### 2.9. Transmission Electron Microscope Observation

The isolated mitochondria pellets were fixed in 2.5% glutaraldehyde at 4°C. The ultrastructure of mitochondrial inner membranes, out membranes, matrix, and cristae was observed using transmission electron microscope [29].

### 2.10. Mitochondrial Respiratory Chain Complex IV Activity Assay

The mitochondrial respiratory chain complex IV (cytochrome c oxidase, COX) activities were determined with a cytochrome c oxidase kit (GENMED). Briefly, a suitable volume of mitochondria solution containing 2 *μ*g protein was added to the assay buffer in each reaction well of 96-well plates. After adding the reaction solution, changes in OD values at 550 nm were recorded immediately using a kinetic program with 10 s interval for 60 s and totally 7 readings were obtained using a Thermo Scientific Varioskan Flash microplate reader [16, 25].

### 2.11. PDHC and KGDHC Activity Measurement

The mitochondrial PDHC and KGDHC activity was determined with corresponding assay kits (GENMED). Each reaction well in 96-well plate contained 25 *μ*g mitochondrial protein and the OD values at 0 min and 5 min after the reaction started were recorded at 600 nm [30].

### 2.12. Statistical Analysis

All data were expressed as the mean ± SEM. Data were analyzed using one-way or two-way ANOVA followed by LSD multiple comparison test with SPSS 17.0 software. *P* < 0.05 was considered statistically significant.

## 3. Results

### 3.1. Effects of Xanthoceraside on the Spatial Memory Impairment Induced by A*β*1-42 in Water Maze Test

**Figure 2:**
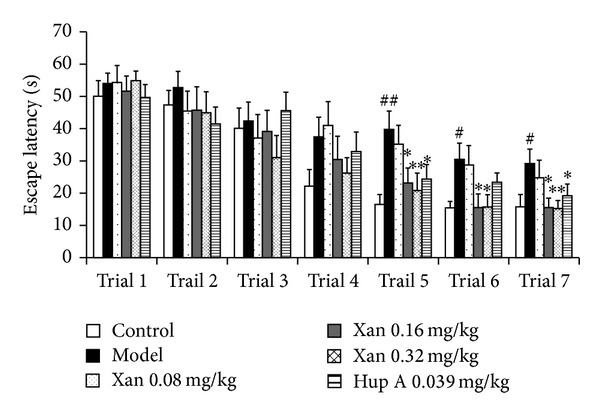
Protective effects of xanthoceraside against A*β*1-42-induced spatial memory impairment in learning period of water maze test in mice. Escape latency to find that the safety platform was recorded during 60 s swimming. The data are presented as means ± SEM (*n* = 9–11). ^##^ *P* < 0.01, ^#^ *P* < 0.05 compared with control group; **P < 0.01, *P < 0.05 compared with model group. Xan: xanthoceraside; Hup A: huperzine A.

**Figure 3:**
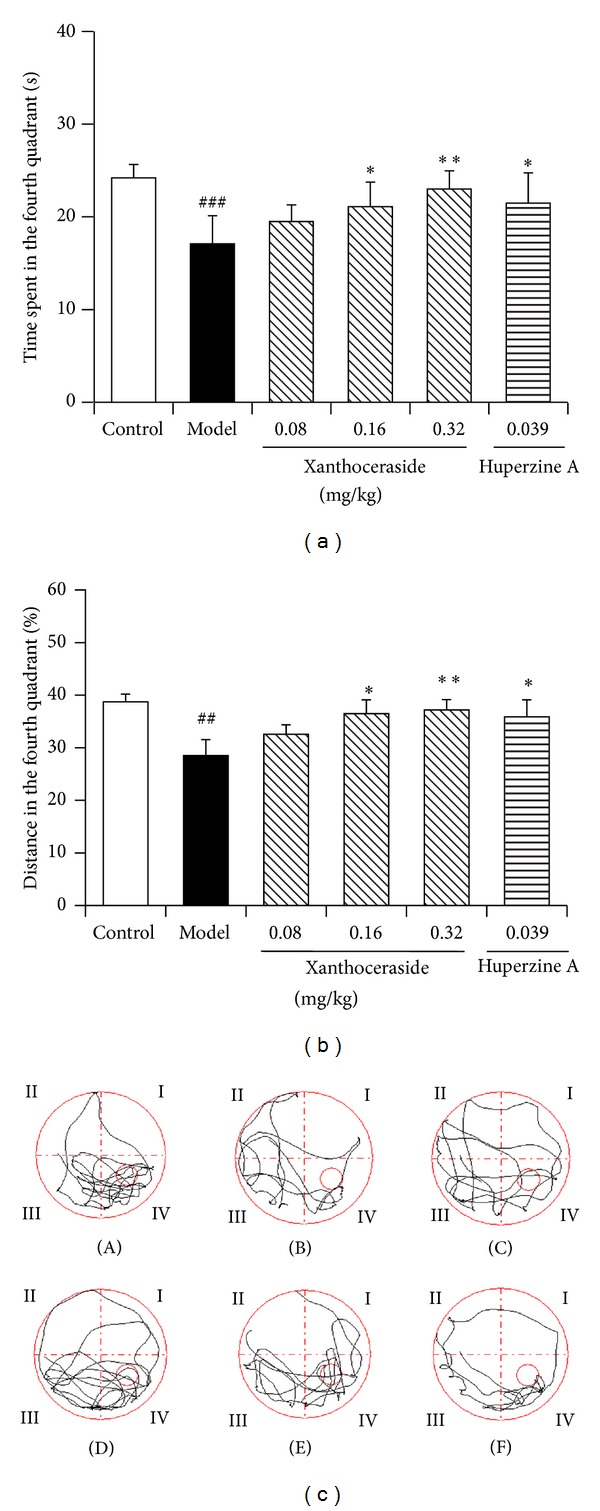
Protective effects of xanthoceraside against A*β*1-42-induced spatial memory impairment in probe test of water maze test in mice. (a) Time spent in the fourth quadrant where the platform has been located. (b) Percentage of the swimming distance in the fourth quadrant where the platform has been located. The data are presented as means ± SEM (*n* = 9–11). ^###^ *P* < 0.001, ^##^ *P* < 0.01 compared with control group; **P < 0.01, *P < 0.05 compared with model group. (c) Characteristic swimming trails of the mice in probe test. (A) Control; (B) model; (C) xanthoceraside 0.08 mg/kg; (D) xanthoceraside 0.16 mg/kg; (E) xanthoceraside 0.32 mg/kg; and (F) huperzine A 0.039 mg/kg.

Water maze test was performed to test the spatial memory ability of the mice. During the training period, A*β*1-42 injected mice consistently spend longer time to find the platform compared to the control group; meanwhile the escape latency of all the mice was getting shorter and shorter as the learning was proceeding. Compared with the A*β*1-42 injected group, treatment with xanthoceraside (0.08–0.32 mg/kg) or huperzine A obviously decreased the escape latency of the mice; moreover, the effects of xanthoceraside were getting significant on the 5th, 6th, and 7th trail, and the improving effects of huperzine A were significant on the 5th and 7th trail (Figure 2). In the probe test, after the platform was removed, the swimming time and the swimming distance in the fourth quadrant of the A*β*1-42 injected mice were significantly shortened compared with the control mice, while mice in the xanthoceraside-treated groups or huperzine A-treated group spent more time and traveled longer length to search in the fourth quadrant compared to the A*β*-injected mice, indicating a better spatial memory ability. The effect of xanthoceraside is in a dose dependent manner and there are statistic significances at 0.16, 0.32 mg/kg xanthoceraside group and 0.039 mg/kg huperzine A group (Figure 3). No significant differences in the swimming speed were observed among the animal groups.

### 3.2. Effects of Xanthoceraside on the Working Memory Impairment Induced by A*β*1-42 in Y-Maze Test

**Figure 4:**
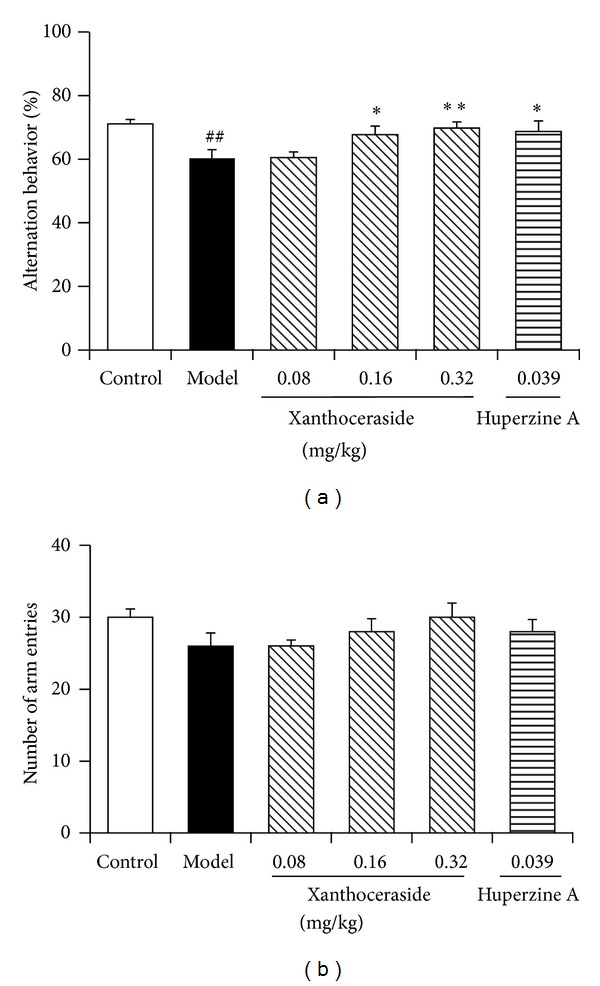
Protective effects of xanthoceraside against A*β*1-42-induced working memory impairment in Y-maze test in mice. Alternation behavior (a) and the number of arm entries (b) were measured during a 5 min session. The data are presented as means ± SEM (*n* = 12). ^##^ *P* < 0.01 compared with control group; **P < 0.01, *P < 0.05 compared with model group.

Y-maze test was performed to determine the working memory ability in mice. A*β*1-42 injected mice showed significant reduction of spontaneous alternation behavior compared with control group. Treatment with xanthoceraside (0.16, 0.32 mg/kg) or huperzine A 0.039 mg/kg significantly attenuated the impairment of spontaneous alternation behavior in A*β*1-42 injected mice. The effect of xanthoceraside was in a dose dependent manner. The total numbers of arm entries showed no difference in all the groups, showing that the treatment did not influence the spontaneous activity of the mice (Figure 4).

### 3.3. Effects of Xanthoceraside on ATP Levels in the Cerebral Cortex and Hippocampus of the Mice Induced by A*β*1-42

**Figure 5:**
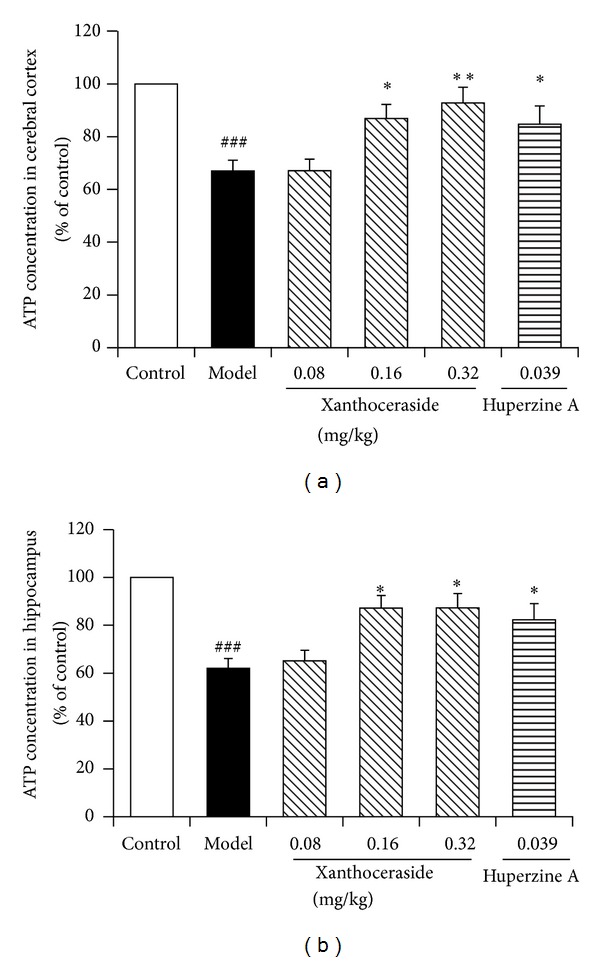
Effects of xanthoceraside on ATP levels in the cerebral cortex and hippocampus of the mice induced by A*β*1-42. (a) The ATP concentration in cerebral cortex. (b) The ATP concentration in hippocampus. The data are presented as means ± SEM (*n* = 7-8). ^###^ *P* < 0.001 compared with control group; **P < 0.01, *P < 0.05 compared with model group.

The cellular ATP levels reflect the functional state of mitochondria, so we tested the ATP levels in cerebral cortex and hippocampus tissue cells of the mice to confirm the mitochondrial functions. The results showed that A*β*1-42 injected mice showed a significant decrease of ATP levels in the cerebral cortex and hippocampus compared with control group, indicating the impaired mitochondrial function. However, treatment with xanthoceraside (0.16, 0.32 mg/kg) or huperzine A 0.039 mg/kg significantly reversed the aberrant decrease of ATP levels in the cerebral cortex and hippocampus (Figure 5), improving the mitochondrial function.

### 3.4. Effects of Xanthoceraside on ROS Levels in the Cerebral Cortex and Hippocampus of the Mice Induced by A*β*1-42

**Figure 6:**
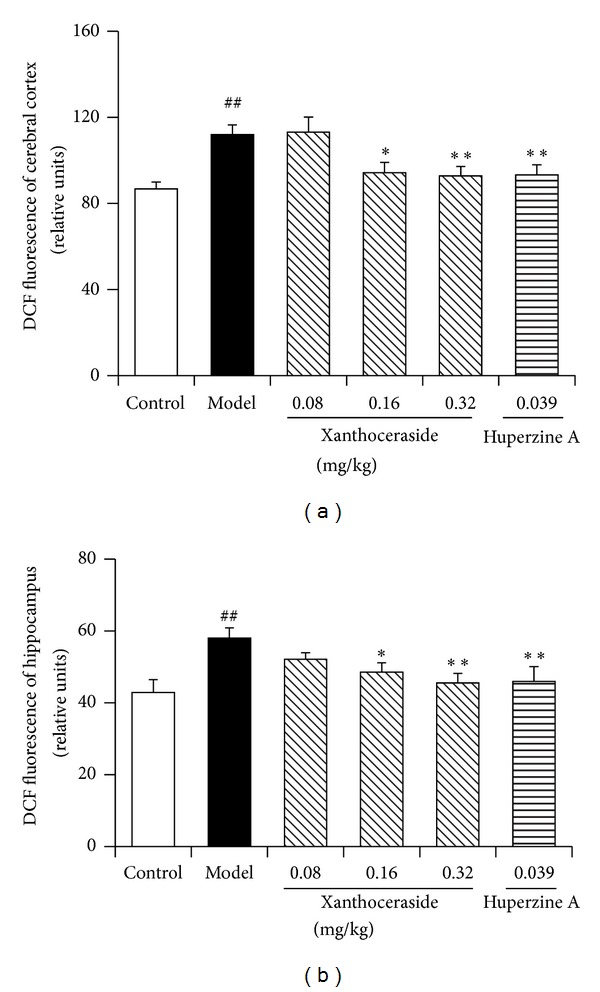
Effects of xanthoceraside on ROS levels in the cerebral cortex and hippocampus of the mice induced by A*β*1-42. (a) The ROS level in cerebral cortex. (b) The ROS level in hippocampus. The data are presented as means ± SEM (*n* = 7-8). ^##^ *P* < 0.01 compared with control group; **P < 0.01, *P < 0.05 compared with model group.

We further determined the ROS levels in the cerebral cortex and hippocampus tissue cells of the mice to confirm the impaired mitochondrial function and the resulting ROS overproduction. It showed that, compared with control group, ROS levels in the cerebral cortex and hippocampus of A*β*1-42 injected mice were significantly increased. Xanthoceraside (0.08–0.32 mg/kg) dose dependently attenuated the increase of ROS levels both in the cerebral cortex and hippocampus induced by A*β*1-42 in mice. Huperzine A group (0.039 mg/kg) showed significant decrease of ROS as well compared with A*β*1-42 injected group (Figure 6).

### 3.5. Mitochondrial Preparations

**Figure 7:**
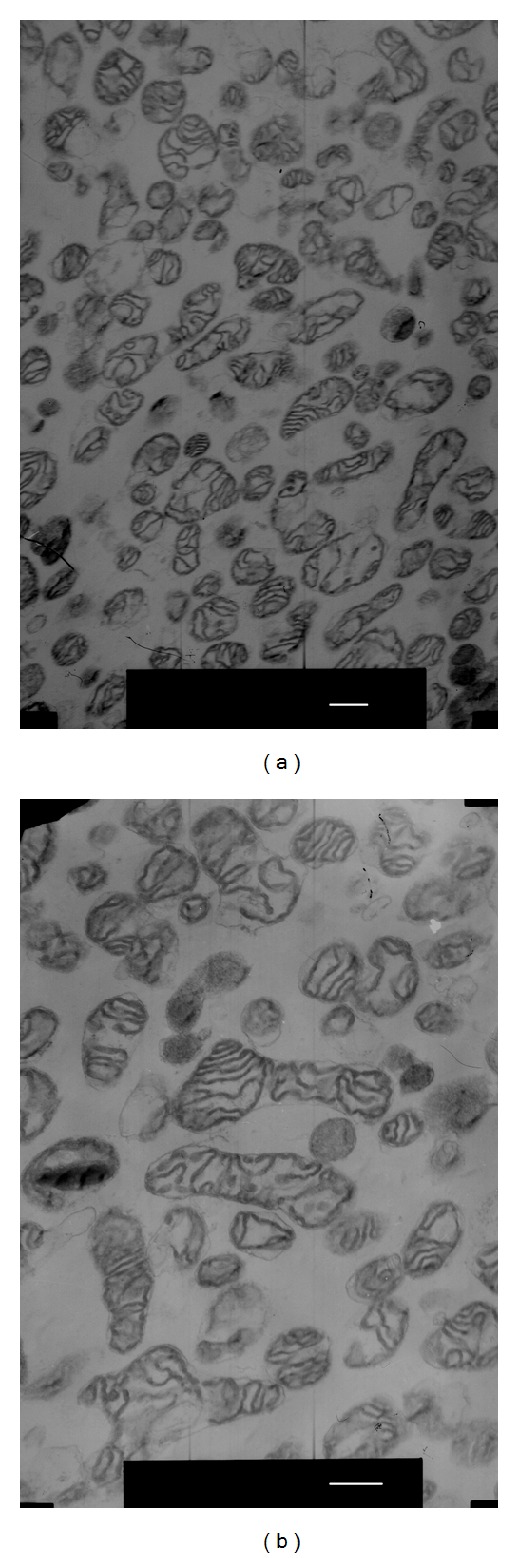
Representative electron micrograph of mice brain mitochondria isolated by the method described in Section 2.8. (a) Mitochondria magnified by 10000 times; (b) mitochondria magnified by 15000 times. Scale bar = 500 nm.

To identify the status of the obtained mitochondria, the ultrastructure morphology was observed by transmission electron microscope. The results showed that almost all the obtained mitochondria exhibited intact mitochondrial membranes, uniform matrix, and a clear structure of cristae, suggesting that the mitochondria used in current study were intact and healthy. Moreover, the view of the photo was filled with mitochondria with very little contamination of synaptosome and other cellular components as shown in Figure 7.

### 3.6. Effects of Xanthoceraside on Mitochondrial Respiratory Chain Complex IV Activity in Isolated Cerebral Cortex Mitochondria of the Mice

**Figure 8:**
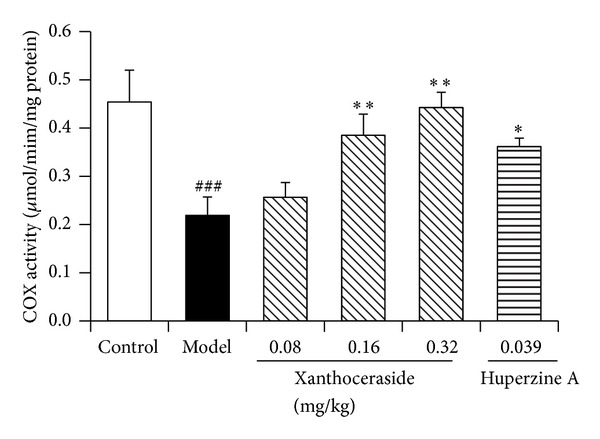
Effects of xanthoceraside on cytochrome c oxidase (COX) activity in isolated cerebral cortex mitochondria of A*β*1-42-induced mice. The data are presented as means ± SEM (*n* = 7-8). ^###^ *P* < 0.001 compared with control group; **P < 0.01, *P < 0.05 compared with model group.

Mitochondrial respiratory chain complex IV (COX) is the key enzyme in the terminal step of mitochondrial electron transport chain. Its activity is critical for mitochondrial respiration, ATP production, and cell survival. Therefore, we tested its activity in the isolated mitochondria of cerebral cortex in mice. The results showed that A*β*1-42 injection led to a significant decrease of COX activity compared with control group. Administration of xanthoceraside (0.08–0.32 mg/kg) dose dependently attenuated the abnormal decrease of COX activity induced by A*β*1-42. Likewise, the decrease of COX activity in A*β*1-42 injection group was also alleviated by the treatment of 0.039 mg/kg huperzine A (Figure 8).

### 3.7. Effects of Xanthoceraside on PDHC and KGDHC Activity in Isolated Cerebral Cortex Mitochondria of the Mice

**Figure 9:**
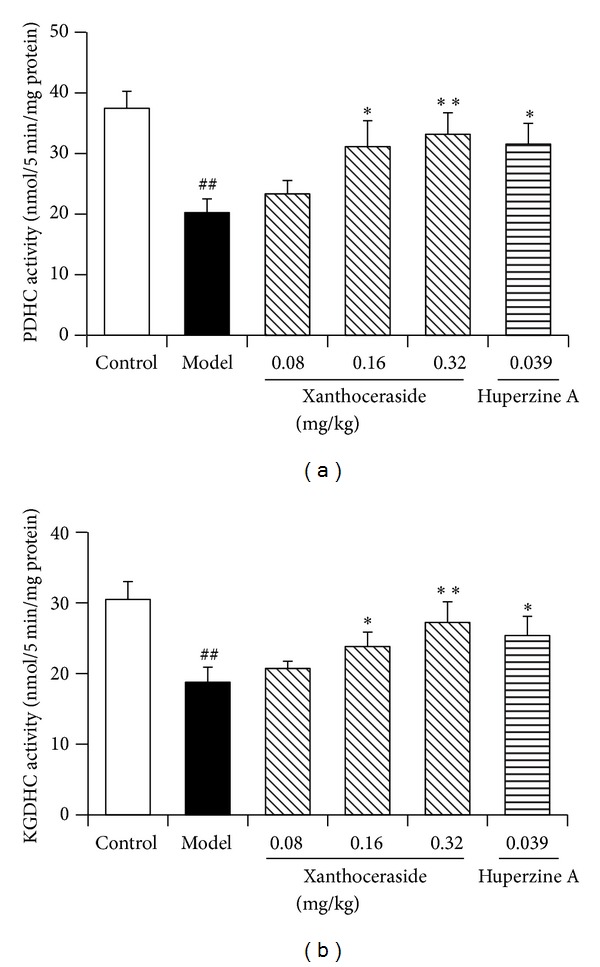
Effects of xanthoceraside on PDHC and KGDHC activity in isolated cerebral cortex mitochondria of A*β*1-42-induced mice. (a) The activity of PDHC and (b) the activity of KGDHC. The data are presented as means ± SEM (*n* = 7-8). ^##^ *P* < 0.01 compared with control group; **P < 0.01, *P < 0.05 compared with model group. PDHC: pyruvate dehydrogenase complex; KGDHC: *α*-ketoglutarate dehydrogenase complex.

PDHC and KGDHC are key matrix enzymes in mitochondrial Kreb's cycle; therefore, we further determined the effects of xanthoceraside on the activity of PDHC and KGDHC. The results showed that A*β*1-42 injected model mice showed significant decrease in both PDHC and KGDHC activity. Xanthoceraside (0.16, 0.32 mg/kg) and huperzine A 0.039 mg/kg significantly reversed the decrease of PDHC and KGDHC activity in isolated cerebral cortex mitochondria of the mice compared with A*β*1-42-injected model group (Figure 9).

## 4. Discussion

Based on the research results, it was recognized that soluble A*β* species especially A*β* oligomers rather than the final plaques may possess more severe neurotoxicity, since it is more correlated to the cognitive deficits in AD patients [31]. Therefore, the animal models obtained after soluble A*β* peptide injection in the brain may be seen as an alternative to transgenic animals to study the AD mechanism and drug development, despite the fact that there is no presence of plaques in the brain of the model animals. Indeed, data showed that i.c.v. administration of incubated A*β* in mice/rats could induce memory deficits, inhibit LTP, and lead to oxidative stress and neuronal loss [32, 33]. Additionally, this model is rather suitable and efficient to test compounds which could act at early stages of AD. Since mitochondrial dysfunction is also considered as the early event of AD, we tended to choose the A*β*1-42 injected (i.c.v.) mice model in the present study. Many researchers identified that 400 pmol or higher doses of A*β*1-42 i.c.v. injection could induce learning and memory deficits in rodents. Therefore, we chose 410 pmol A*β*1-42 as inducing dose in the model group, which is employed before by other researchers and also proved reliable in our previous studies [23, 33]. Water maze test and Y-maze test were used to measure the learning and memory ability of the mice. The results clearly demonstrated that A*β*1-42 could significantly induce the learning and memory deficits which is consistent with the results of other researchers [23, 33]. And xanthoceraside significantly attenuated the learning and memory deficits induced by A*β*1-42; however, it did not affect the spontaneous activity in mice (data not shown).

As the positive control, huperzine A was shown to improve learning and memory deficits in our present study. Huperzine A is a potent reversible inhibitor of acetylcholinesterase and has been proved to improve learning and memory performance in AD patients in China [34, 35]. Recent studies indicated its neuroprotective effects including inhibition of A*β* neurotoxicity [35–37], which is likely to involve prevention of oxidative stress and improvement of mitochondrial function [38, 39]. Therefore, we chose huperzine A as the positive control and our results further proved its effects on alleviating mitochondrial dysfunction, inhibiting oxidate stress, and attenuating energy depletion.

Our previous studies indicated that xanthoceraside could reverse A*β*-induced ROS overproduction and inhibit A*β*-induced apoptosis in SH-SY5Y cells [40] and it also had the effect of antioxidative stress and antiapoptosis in A*β*-injected mice model (to be published), which could be the underlying mechanism of xanthoceraside to improve learning and memory impairment. However, we have not found the inhibitory effects of xanthoceraside on acetylcholinesterase (AchE) in the hippocampus of control mice (to be published). Therefore, the mechanisms of xanthoceraside on improving learning and memory deficits need further elucidation. Mitochondrial dysfunction has been found in many diseases including AD, PD, and cancer with different mechanisms involved [41, 42]. Researchers have found the direct interaction between A*β* and mitochondria; for example, A*β* and ABAD interact in brain mitochondria of AD patients and transgenic mice overexpressing ABAD leading to mitochondrial dysfunction [7, 43]; the causality of A*β* and COX deficiency are also under debate by now. Therefore, based on our previous study, we proposed that xanthoceraside may possibly attenuate mitochondrial dysfunction induced by A*β*1-42, which may contribute to its antioxidative stress and antiapoptosis effects. The results of present study would help to further expound the mechanisms of xanthoceraside on improving learning and memory deficits in AD.

One of the most important roles of mitochondria is to supply energy to the cells with ATP as energy molecules. The brain has very high energy requirement for neurotransmission and maintaining calcium homeostasis; therefore, the ATP depletion in the brain has devastating influence on the neural activities [13]. Our results showed that xanthoceraside could significantly attenuate the ATP depletion in the cerebral cortex and hippocampus compared with model mice induced by A*β*1-42, improving the mitochondrial dysfunction. Some studies reported that the interaction between A*β* and ATP synthesis may result in ATP deletion [44]. Furthermore, a subsequent study reported that A*β* could decrease the activity of cytochrome c oxidase (COX) and inhibit the mitochondrial respiratory function, finally, leading to the ATP deletion and ROS overproduction.

In the present study, we also found that A*β* could increase the ROS level in the cerebral cortex and hippocampus of model mice and xanthoceraside had alleviative effects. Many studies prompted that oxidative stress occurs in very early stages of AD ahead of massive A*β* deposits. Oxidative stress causes lipid oxidation, protein modification, and DNA mutation and therefore generates a huge damage to many organelles including mitochondria [45, 46]. Several lines of evidence suggest that increased mitochondrial ROS are responsible for changes in mitochondrial structure and function including mitochondrial membrane damage, mitochondrial fission/fusion imbalance, and mitochondrial enzyme inhibition [47]. As the natural by-product of mitochondrial electron transfer, ROS was mostly produced in mitochondria. ROS deriving from mitochondria further damage mitochondria to form a vicious cycle, which could contribute to the AD progression. The decrease of ROS caused by xanthoceraside could be a contributory mechanism to its neuroprotective effects.

It was pointed out that increased ROS and the resulted oxidative stress damage are inevitable outcomes of mitochondrial respiratory deficits [46, 48]. Research data showed that mitochondrial respiratory chain complex IV (cytochrome c oxidase, COX) activity was decreased in AD transgenic mice and synthetic A*β*1-42 specifically inhibited the COX activity in a dose dependent manner [49]. In fact, a reduced activity of COX has been reported in different brain regions of AD patients. In the present study, we found that A*β*1-42 led to a significant decrease of COX activity and administration of xanthoceraside attenuated the abnormal decrease of COX activity induced by A*β*1-42, which could be one of the possible reasons for xanthoceraside to lower the ROS level and improve the mitochondrial function. However, we should mention that the massive oxidative stress in AD not only is due to the ROS overproduction but also is due to the decrease of ROS elimination by mitochondrial antioxidant defense system [47, 50]. Our previous studies showed that xanthoceraside could also significantly increase the activity of catalase and the ratio of GSH/GSSG impaired by A*β*1-42, elevating the cellular antioxidant ability. We certainly cannot exclude other possible mechanisms which participate in the oxidative stress induced by A*β* and the possible effects of xanthoceraside.

Several positive emission tomography (PET) scan studies revealed reduced glucose metabolism in the brains of AD patients, indicating the decreased glucose metabolism [9]. Many biochemical studies found the decline of several mitochondrial enzymes such as PHC and KGDHC in the brain of AD patients. PHC is the key rate-limiting enzyme to initiate the mitochondrial Kreb's cycle and the reaction catalyzed by KGDHC is the crucial spot to produce energy in Kreb's cycle. In fact, they serve as the regulatory switch coupling glucose utilization to oxidative phosphorylation [51]. In present study, we found that xanthoceraside could reverse the decrease of PDHC and KGDHC activity in isolated cerebral cortex mitochondria of the mice induced by A*β*1-42. It was reported that A*β* may induce the decrease of PDHC and KGDHC activity by the overproduction of ROS. The exact mechanism needs further studies.

To exclude the disturbance of cytoplasmic protein and other cellular components, we used isolated mitochondria for the COX, PDH, and KGDHC detection. The mitochondria were prepared using Percoll density gradient centrifugation method developed by Sims and Anderson [26–28], which is wildly accredited to acquire highly metabolically mitochondrial fractions with good respiratory coupling and little contamination with synaptosomes or myelin. We observed the ultrastructure of obtained mitochondria by the electron microscope, and the preparations of cerebral cortex mitochondria in the present study appeared homogeneous and intact with very little contaminations. All of which ensured the reasonability of our research.

Altogether, these results showed that xanthoceraside can ameliorate the working memory and spatial learning and memory impairments induced by A*β*1-42 probably through alleviating mitochondrial dysfunction, inhibiting oxidate stress, and attenuating energy depletion. Actually, more work needs to be done to elucidate the mechanism for xanthoceraside to improve mitochondrial dysfunction, such as the effects on mitochondrial dynamic (balance of mitochondrial fusion/fission), interaction of A*β* and ABAD, and mitochondrial axonal transport [52–54].

Our preliminary toxicological assessment showed that xanthoceraside has no adverse effect on general behavior and motor coordination ability and no synergistic effect with pentobarbital sodium in mice (data not shown). At dose of 400 times of effective dose, xanthoceraside did not cause the death of the tested mice and no macroscopic changes were found in the main viscera organs in mice. In addition, the oil extracted from XSB fruits used to be cooking oil, all suggesting that xanthoceraside has a good safety profile. Taken together, xanthoceraside has the potential to be a competitive candidate drug for the treatment of AD.
